# Comparative Microbiome Signatures and Short-Chain Fatty Acids in Mouse, Rat, Non-human Primate, and Human Feces

**DOI:** 10.3389/fmicb.2018.02897

**Published:** 2018-11-30

**Authors:** Ravinder Nagpal, Shaohua Wang, Leah C. Solberg Woods, Osborne Seshie, Stephanie T. Chung, Carol A. Shively, Thomas C. Register, Suzanne Craft, Donald A. McClain, Hariom Yadav

**Affiliations:** ^1^Department of Internal Medicine-Molecular Medicine, Wake Forest School of Medicine, Center for Diabetes, Obesity and Metabolism, Winston-Salem, NC, United States; ^2^Department of Microbiology and Immunology, Wake Forest School of Medicine, Center for Diabetes, Obesity and Metabolism, Winston-Salem, NC, United States; ^3^Department of Physiology and Pharmacology, Wake Forest School of Medicine, Winston-Salem, NC, United States; ^4^Diabetes, Endocrinology and Obesity Branch, National Institutes of Diabetes, Digestive and Kidney Diseases, National Institutes of Health, Bethesda, MD, United States; ^5^Department of Pathology-Comparative Medicine, Wake Forest School of Medicine, Winston-Salem, NC, United States; ^6^Department of Internal Medicine-Gerontology and Geriatric Medicine, Wake Forest School of Medicine, Winston-Salem, NC, United States; ^7^Department of Endocrinology and Metabolism, Wake Forest School of Medicine, Winston-Salem, NC, United States

**Keywords:** microbiome, short-chain fatty acids, microbiota, mice, rat, non-human primate, metabolites, monkey

## Abstract

Gut microbiome plays a fundamental role in several aspects of host health and diseases. There has been an exponential surge in the use of animal models that can mimic different phenotypes of the human intestinal ecosystem. However, data on host species-specific signatures of gut microbiome and its metabolites like short-chain fatty acids (SCFAs; i.e., acetate, propionate, and butyrate) and lactate in these models and their similarities/differences from humans remain limited, due to high variability in protocols and analyses. Here, we analyze the fecal microbiota composition and the fecal levels of SCFAs and lactate in three of the most-widely used animal models, i.e., mice, rats, and non-human primates (NHPs) and compare them with human subjects, using data generated on a single platform with same protocols. The data show several species-specific similarities and differences in the gut microbiota and fecal organic acids between these species groups. Based on β-diversity, the gut microbiota in humans seems to be closer to NHPs than to mice and rats; however, among rodents, mice microbiota appears to be closer to humans than rats. The phylum-level analyses demonstrate higher *Firmicutes–Bacteroidetes* ratio in humans and NHPs vs. mice and rats. Human microbiota is dominated by *Bacteroides* followed by *Ruminococcaceae* and *Clostridiales*. Mouse gut is predominated by members of the family *S24-7* followed by those from the order *Clostridiales*, whereas rats and NHPs have higher abundance of *Prevotella* compared with mice and humans. Also, fecal levels of lactate are higher in mice and rats vs. NHPs and humans, while acetate is highest in human feces. These data of host species-specific gut microbiota signatures in some of the most widely used animal models in context to the human microbiota might reflect disparities in host factors, e.g., diets, genetic origin, gender and age, and hence call for prospective studies investigating the features of gut microbiome in such animal models by controlling for these host elements.

## Introduction

The gut microbiome plays a key role in various aspects of human health including nutrient digestion and metabolism, development and maturation of immune system, protection from infections, nervous system development, and has widespread influence beyond the gastrointestinal tract (Blanton et al., 2016; Kho and Lal, 2018). Numerous clinical studies have demonstrated the association of gut microbes with a wide array of cardiometabolic and chronic diseases including obesity, type 2 diabetes, atopic diseases, cardiovascular diseases, hypertension, anxiety, depression, bowel diseases, diarrhea, constipation, and brain diseases including Parkinson’s, Alzheimer’s, and others (Bakhtiar et al., 2013; Cojocaru and Chicos, 2014; Althani et al., 2016; Coit and Sawalha, 2016; Scher et al., 2016; Davis and Bajaj, 2017; Gundogdu and Nalbantoglu, 2017; Ipci et al., 2017; Ruiz-Rodriguez and Rello, 2017; Shivaji, 2017; Blutt et al., 2018; Davidson and Epperson, 2018). However, given the practical and ethical complexity of performing invasive sampling procedures in human subjects, high inter-individual variation in the diets and in the gut microbiomes of humans, and relative ease of using animals with controlled diets for large scale mechanistic and genotypic research studies, different types of animals models including rodents (mice, rats, guinea pigs, and hamsters), rabbits, pigs, zebra fish, and non-human primates (NHPs; e.g., macaques and vervet monkeys) have been developed and are frequently used to investigate the multiple dynamics of host–microbiome interactions (Gootenberg and Turnbaugh, 2011; Tlaskalova-Hogenova et al., 2014; Amato et al., 2015; National Academies of Sciences et al., 2018; Nagpal et al., 2018). Among these, rodents – specifically mice and rats – remain the most-widely used models for studying the dynamics of host–gut microbiome interaction in host nutrition and disease development and are convenient models for exploring avenues for developing novel microbial therapies. There has also been an increasing surge in the use of germ-free mice and rats to perturb, orchestrate, and elucidate host–microbiome interplay with a magnitude of experimental paradigms not feasible in humans (Faith et al., 2011). While rodent models are widely used for economic and practical reasons, NHPs are increasingly being used to understand the host–microbiome association in contexts to host genetics, nutrition, environment, and various disease phenotypes (Shively et al., 2009, 2015; Bauer et al., 2011; Amato et al., 2015; Nagpal et al., 2018) due to their genetic and physiological closeness to humans.

However, understanding and interpreting the information obtained from such model systems in relation to the human milieus importantly requires knowledge of major similarities and dissimilarities. This is particularly important given the large differences in various arrays of genetics, intestinal anatomy, gut physiology, enteric immune network, metabolism, dietary behavior, and others (all of which may also correspond to microbiome differences) not only between humans and different animal species. In addition, among animal models such as mice, rats, guinea pigs, hamsters, rabbits, swine, monkeys, and others generally used to mimic the humanized gastrointestinal tract settings. Few independent studies have reported the features of gut microbiome configuration in different animal species (Nguyen et al., 2015); however, studies comparing the gut microbiome composition in different animal species with that in humans are illusive. Comparative studies of the gut microbiome in multiple host species have generally been performed by acquiring the data from different database portals or animal samples from different locations and might not provide clear-cut information due to confounding effects of disparities in geographical settings, experimental protocols, and sequencing analysis approaches. The current study utilized the same analytical platform to examine the gut microbiota composition and the fecal levels of SCFAs and lactate in some of the most-commonly used animal models including mice, rats, and NHPs, and were then compared with human subjects.

## Materials and Methods

The study included in-bred C57BL/6 mice (male; *n* = 24; mean age 14 weeks; diet: normal chow *ad libitum*); outbred NIH heterogeneous stock rats (Hansen and Spuhler, 1984; Woods and Mott, 2017) (male; *n* = 17; 18 weeks of age after 12 weeks on a low fat (LF) or high fat (HF) diet: 8 rats were on a HF diet (60% kcal from fat) and 9 rats were on a LF diet (10%kcal from fat) *ad libitum*); apparently healthy adult NHPs (cynomolgus macaques [*Macaca fascicularis*]; female; *n* = 25; mean age: 8.8 years; maintained on a human-style Mediterranean or Western diet for the last 34 months; (Nagpal et al., 2018); and human subjects (*n* = 25; female/male 18/7; mean age: 39.3 years; enrolled at Wake Forest School of Medicine and National Institutes of Diabetes and Digestive and Kidney Diseases, National Institutes of Health for on-going studies). Rat diets were purchased from Research Diets (LF: D12492; HF: D12450J). All mice and rats were maintained at the Wake Forest Biotech Place Animal Resource Program facility, and the NHPs were maintained at the Wake Forest University Primate Center. To collect the fecal samples, mice were placed in an empty sterile cage for 5–10 min and freshly dropped fecal pellets were collected aseptically into sterile tubes using sterile tweezers. Rats were euthanized by decapitation and fecal body was collected from rectum and placed into sterile tubes. The monkeys were euthanized with pentobarbital (60 mg/kg), and fecal samples of rectal/anal contents were collected at the time of necropsy and immediately placed in sterile tubes under aseptic conditions. Notably, mice, rats, and monkeys were parts of different projects and were subsequently euthanized for the collection of blood, organs, tissues, etc. in accordance with respective approved protocol by institutional review board (IRB) and institutional animal care and use committee (IACUC). Human subjects were asked to collect freshly voided fecal samples into the fecal collection container, place the samples immediately in a cooling box containing refrigerants, transport at earliest possible (within 24 h) to the laboratory where these were stored immediately after collection at -80°C until further processing. All animal manipulations and human sample collections were performed according to the guidelines of state and federal laws, the US Department of Health and Human Services, and the Animal Care and Use Committee of Wake Forest University School of Medicine.

16S rRNA gene amplification and sequencing was done as per our previously reported method (Nagpal et al., 2018). Briefly, genomic DNA was extracted from approximately 200 mg of feces by using Qiagen DNA Stool Mini Kit (Qiagen, Valencia, CA, United States), with a slight modification, i.e., using lysis temperature of 95°C instead of 75°C for efficient lysis and DNA yield of Gram-positive bacteria. Each batch of DNA extraction included a negative control wherein nuclease-free water (Invitrogen, Eugene, OR, United States) was used in place of feces. All controls were processed, amplified, and sequenced together with samples. The hypervariable region V4 of the bacterial 16S rRNA gene was amplified using the primers 515F (barcoded) and 806R in accordance with the Earth Microbiome Project protocol (Caporaso et al., 2012), with minor modifications as described in our previous study (Nagpal et al., 2018). The amplicons were purified using AMPure XP beads (AMPure^®^ XP magnetic purification beads, Beckman Coulter, Inc., Brea, CA, United States), and the purified PCR products were quantified on Qubit-3 fluorimeter (Invitrogen). Equal amounts of purified products were pooled; the pool was quantified again and normalized to 4 nM, then denatured and diluted to 8 pM for sequencing on an Illumina MiSeq sequencer (Miseq reagent kit v3; Illumina, San Diego, CA, United States). The sequences generated were de-multiplexed, quality-filtered, clustered and analyzed using QIIME (Quantitative Insights into Microbial Ecology; version 1.9.1) (Caporaso et al., 2010). After establishing operational taxonomic units (OTUs) at 97% identity, a total of 32,49,725 reads (mean ± SEM = 35711 ± 3383) were generated after filtering. To avoid any bias of different sequencing depth, the OTU table was rarefied to the lowest number of sequences per sample for generating alpha-diversity metrics within QIIME. The raw data of alpha-diversity metrices and OTU abundance in each sample is provided in Supplementary Table S1.

Bacterial taxonomy assignment and diversity analysis was calculated within QIIME using default settings to compare the bacterial species richness between the four species groups. Alpha diversity (observed OTUs, Chao1, PD_Whole_Tree, and Shannon) indices were computed using core_diversity_analysis.py script. Beta diversity was computed within QIIME using weighted and unweighted UniFrac distance metrics (Lozupone et al., 2011). Principal component analysis (PCA) was executed to determine the pattern of overall microbiota composition in different groups. PCA plots were visualized using EMPeror version 0.9.3-dev. OTUs with abundance lower than 1% were excluded from downstream analyses. Differences in beta-diversity were tested by permutational multivariate analysis of variance (PERMANOVA), a permutation-based multivariate analysis of variance to a matrix of pairwise distance to partition the inter-group and intra-group distance. Linear discriminant analysis (LDA) analysis and LDA-based cladograms were executed on the top 100 OTUs using the LDA effect size (LefSe) algorithm, as described previously (Segata et al., 2011). Hierarchical clustering heat-maps based on average linkage on Euclidean distance were prepared in R using the “ggplots” (version 3.0.1) library.

To measure SCFAs and lactate, an approximately 100 mg aliquot of feces was aseptically mixed with 900 μL sterile PBS buffer (pH 7.4) in a sterile tube and vortexed for 1 min or until uniformly suspended. The suspension was centrifuged at 12,000 *g* for 10 min and the supernatant was filtered through a 0.45 μm membrane filter. Concentrations (μmol/gram of fecal sample) of lactate, acetate, propionate, and butyrate were determined using a high-performance liquid chromatography (Waters-2695 Alliance HPLC system, Waters Corporation, Milford, MA, United States) with DAD detector at 210 nm, equipped with a Aminex HPX-87H column (Bio-Rad Laboratories, Hercules, CA, United States). Sample (10 μL) was injected and H_2_SO_4_ (0.005 N) was used to elute the column with a flow rate of 0.6 mL/min at 35°C.

The bacterial diversity and abundance and the fecal SCFAs and lactate levels between the four groups were compared by using non-parametric analyses in R (version 3.4.3^1^). Statistically significant inter-group differences were calculated using the Kruskal–Wallis test followed by Dun’s *post hoc* analysis with Bonferroni correction. Results are expressed as mean ± standard deviation. Unless otherwise stated, *P* < 0.05 was considered statistically significant.

## Results

### Microbiome Beta- and Alpha-Diversity Indices Among Host Species

**FIGURE 1:**
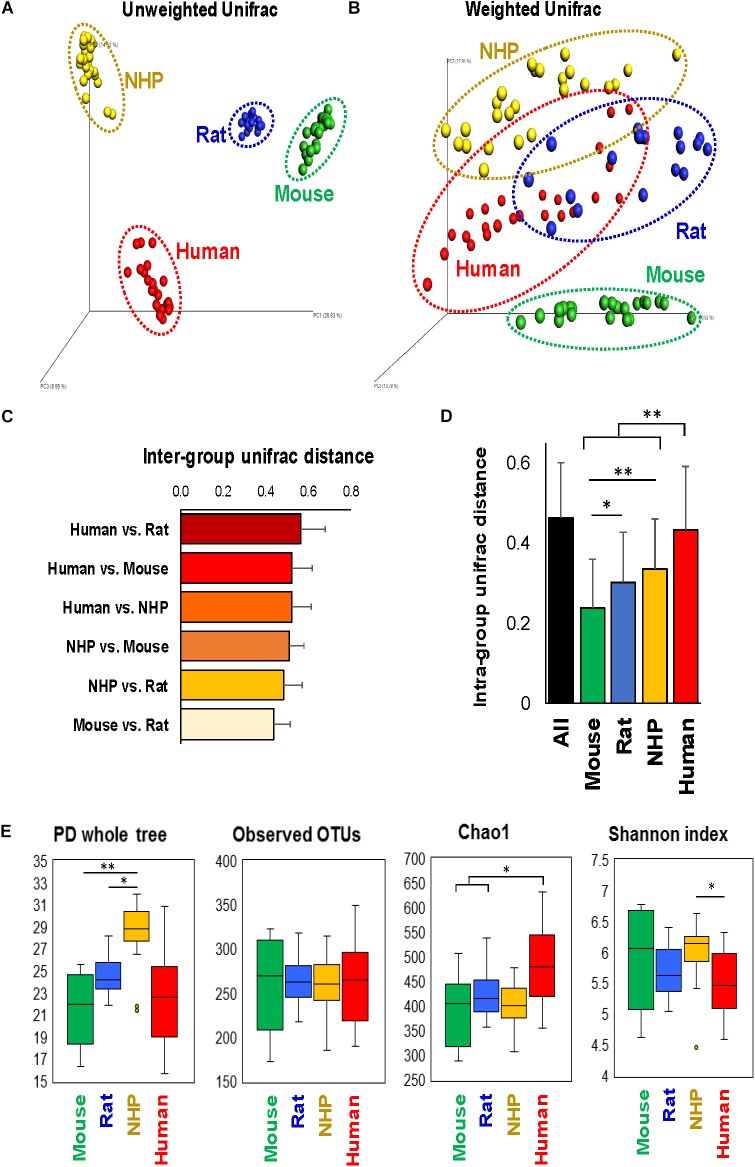
Configuration of gut microbiome diversity in mice, rats, non-human primates (NHP), and human subjects. PCoA analysis of unweighted **(A)** and weighted **(B)** UniFrac distance representing beta-diversity of the gut microbiota in mice, rats, non-human primates, and human subjects. Inter-group **(C)** and intra-group **(D)** weighted UniFrac distance metrics between and within, respectively, mice, rats, non-human primates, and humans (mean ± SD). **(E)** Indices representing alpha-diversity of the gut microbiota in mice, rats, non-human primates, and human subjects. ^∗^*P* < 0.05, ^∗∗^*P* < 0.001 (Kruskal–Wallis test followed by a Dunn’s post-test with Bonferroni correction).

The analysis of overall β-diversity of gut microbiota signatures in the four species in terms of unweighted (qualitative) as well as weighted (quantitative) UniFrac distance produced four distinct clusters and thus suggested that the gut microbiota in these four species groups has a different composition (unweighted) and structure (weighted) (Figures 1A,B); however, the clusters were more isolated in terms of unweighted UniFrac distance as compared to weighted distance. Further analysis of inter-group UniFrac distances between these four groups revealed the highest distance between the microbiota in humans vs. rats, followed by humans vs. mouse and NHPs, NHPs vs. mouse and rat, with least distance between mouse and rat gut microbiota (Figure 1C). In terms of intra-group UniFrac distance, the gut microbiota in human subjects demonstrated the greatest range, followed by NHPs, rats and mice (Figure 1D). The analysis of α-diversity indices demonstrated the highest phylogenetic diversity in NHPs as compared to that in the other three species, although the number of observed OTUs remained comparable between all four species (Figure 1E). The Chao1 index (OTU richness) was higher in humans vs. mice, rats and NHPs, whereas the Shannon index (OTU biodiversity in terms of OTU richness, abundance and evenness) was higher in NHPs vs. humans but not versus/or among other groups (Figure 1E).

### Differences Between Host Species Are Represented at the Phylum, Family, and Genus Levels

**FIGURE 2:**
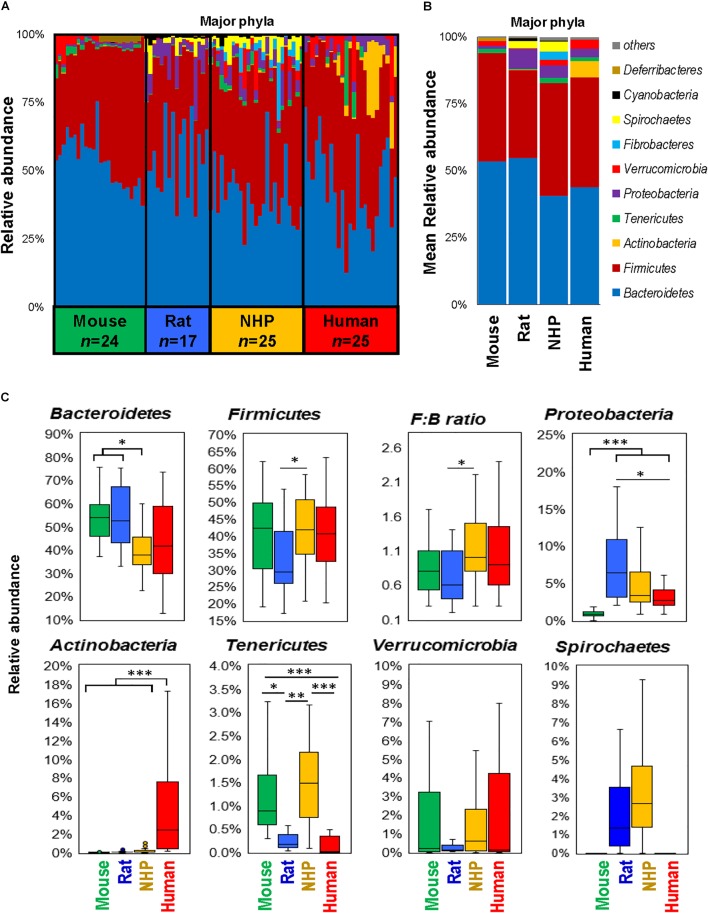
Differences in the abundance of major gut bacterial phyla in mice, rats, non-human primates (NHP), and human subjects. Bar graphs showing sample-wise **(A)** and mean **(B)** relative abundance of the major phyla in mice, rats, non-human primates, and human subjects. **(C)** Box-plots representing the mean relative abundance of major phyla detected in mice, rats, non-human primates, and human subjects. ^∗^*P* < 0.05, ^∗∗^*P* < 0.001, ^∗∗∗^*P* < 0.0001 (Kruskal–Wallis test followed by a Dunn’s post-test with Bonferroni correction).

The phylum-level analysis also revealed several differences between different species in terms of types as well as the abundance of several phyla (Figures 2A,B). While the gut microbiota in mice and rats appeared to be dominated by *Bacteriodetes* followed by *Firmicutes*, NHP and human microbiota composition demonstrated either comparable or slightly higher abundance of *Firmicutes* vs. *Bacteroidetes* (Figures 2A–C). *Actinobacteria* were the highest in humans whereas they remained hardly detectable in the other three species. The abundance of *Proteobacteria* was the lowest in mice and highest in rats. The abundance of *Tenericutes* was comparable between mice and NHPs and was markedly lower in rats and humans. *Verrucomicrobia* did not show any significant inter-group difference but was numerically very low in rats as compared to mice, NHPs and humans whereas *Spirochaetes* was comparable between rats and NHPs but undetectable in mice and humans (Figures 2A–C).

**FIGURE 3:**
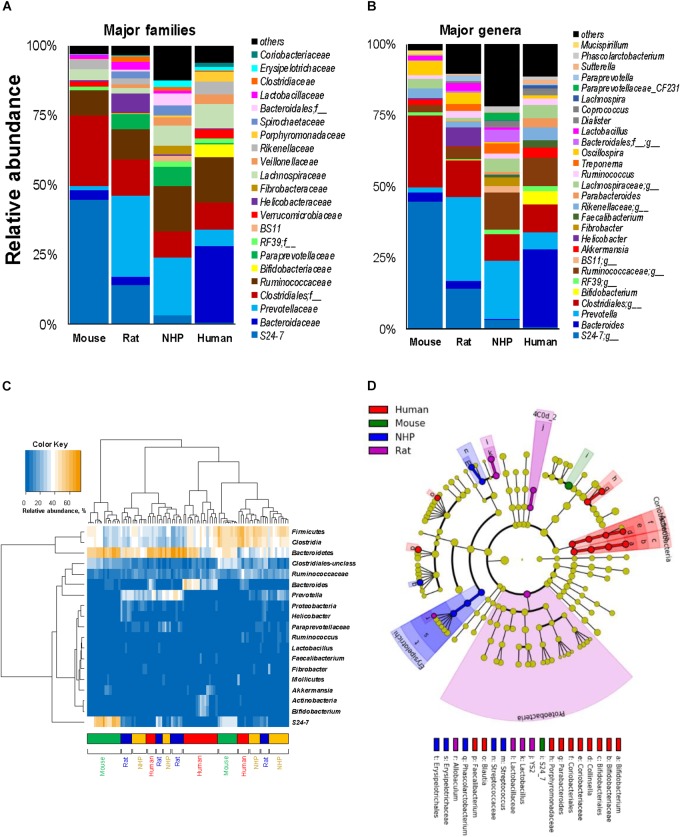
Relative abundance of major gut bacterial families and genera in mice, rats, non-human primates (NHP), and human subjects. Bar graphs representing the relative abundance of major families **(A)** and genera **(B)** detected in mice, rats, non-human primates, and human subjects. **(C)** Heap-map illustrating the hierarchical clustering of top 20 OTUs in mice, rats, non-human primates, and human subjects. **(D)** Linear discriminant analysis (LDA) effect size (Lefse) cladogram representing the unique bacterial signatures identified in mice, rats, non-human primates, and human subjects.

In line with the phylum-level data, the analyses at the family- and genus-level also showed that while many families and corresponding genera are common in the gut of mice, rats, NHPs and human subjects, their abundance vary greatly between different host species (Figures 3A,B). Similar patterns were seen at the class- and order-level (Supplementary Figure S1). At the same time, there were several taxa that were prevalent in some but rarely detected in other host species. For instance, the family *S24-7* (and an unclassified genus of this family) was the predominant *Bacteroidetes* member in mice; whereas in rats and NHPs, the phylum *Bacteroidetes* was represented mainly by *Prevotellaceae* and *Prevotella*. In human subjects, the family *Bacteroidaceae* and the genus *Bacteroides* were the predominant *Bacteroidetes* members followed by *Prevotellaceae* and *Prevotella*, respectively (Figures 3A,B). *Firmicutes* in mice comprised predominantly of OTUs belonging to the order *Clostridiales* whereas in NHPs and humans, the family *Ruminococaceae* (and the genus *Ruminococcus*) was the most abundant group among *Firmicutes*. OTUs belonging to *Bifidobacterium, Faecalibacterium, Coriobacteria*, and *Porphyromonadaceae* were higher in humans compared to all animal species. NHPs had significantly higher abundance of OTUs belonging to *Erysipelotrichaceae, Fibrobacter, Treponema, Paraprevotellaceae* and an unclassified OTU from *Bacteroidales* compared to the other three groups. Rats had relatively higher abundance of *Prevotella*, *Helicobacter*, and *Lactobacillus*. The abundance of *Lactobacillus*, an otherwise important and highly prevalent member of the human gut, was the highest in rats and higher in rodents compared to primates (Figure 3B). *Akkermansia* (major member of the family *Verucomicrobiaceae*) was high in mice and humans but rarely detected in rats and NHPs. These patterns were also observed when the top 20 OTUs detected in these species were subjected to hierarchical heat-map clustering analysis. Mouse samples demonstrated two clusters represented by the dominance of either *Bacteroidetes* (mainly the family *S24-7*) or *Clostridia*, whereas the human samples clustered mainly on the basis of the prevalence of *Bacteroides*, *Clostridia, Bifidobacterium*, and *Akkermansia* (Figure 3C). The clusters generated from rat and NHP samples remained dispersed and overlapped with each other as well as with some human samples (Figure 3C). These signatures were further corroborated by LDA effect size (Lefse) analysis of major OTUs detected in these four species, that produced OTUs that were present in higher abundance in one species versus all of the other three species (Figure 3D and Supplementary Figure S2).

**FIGURE 4:**
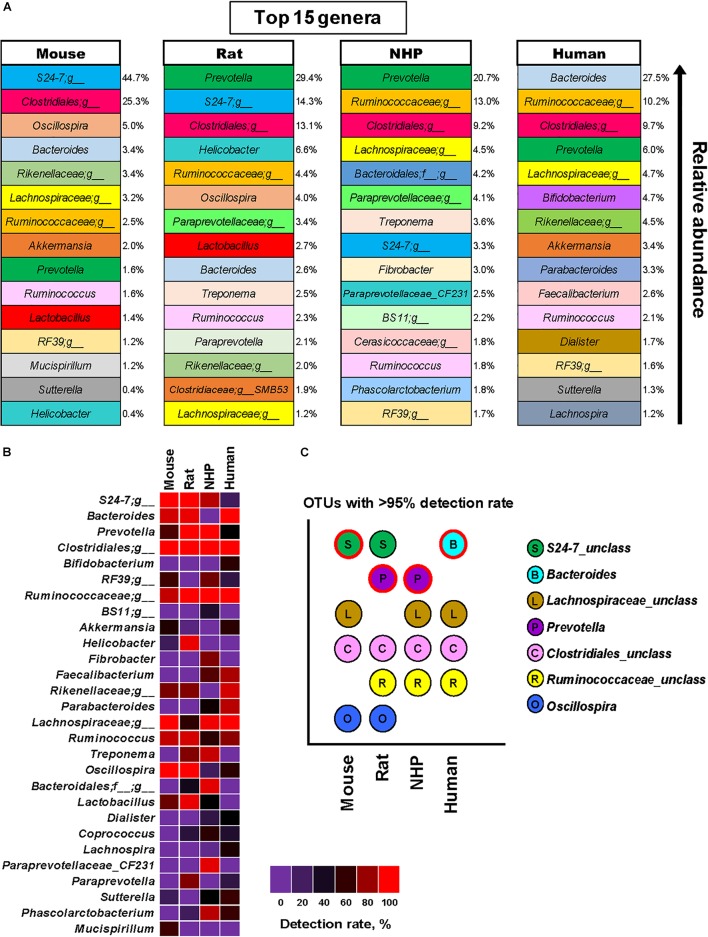
Predominant and prevalent gut bacterial genera in mice, rats, non-human primates (NHP), and human subjects. **(A)** Top 15 genus-level OTUs and their mean relative abundance in mice, rats, non-human primates, and human subjects. **(B)** Detection rate (prevalence, %) of major genus-level OTUs in mice, rats, non-human primates, and human subjects. **(C)** OTUs detected in at least 95% of samples each from mice, rats, non-human primates, and human subjects (the OTU with the highest mean relative abundance in each species group is indicated with red outline).

Based on these analyses, we were able to identify the top 15 genus-level OTUs detected in these four species, as illustrated in Figure 4A showing the major genera in mice, rats, NHPs and humans in terms of the relative abundance. Figure 4B demonstrates some of the major OTUs on the basis of their detection rate (prevalence, %) in these species. In line with the results of relative abundance (Figure 4A), the OTUs belonging to the family *24-7*, the order *Clostridiales*, the family *Lachnospiraceae*, and the genus *Oscillospira* were also found to be the most prevalent groups (detection rate 100%) followed by *Bacteroides* (88%), *Ruminococcus* (83%), and *Prevotella* (50%) in mice (Figure 4C). Rats had the highest detection rate of OTUs belonging to the genus *Prevotella*, the order *Clostridiales*, the family *24-7, Ruminococcaceae* and *Oscillospira* (detection rate 100%) followed by *Bacteroides*, *Helicobacter, Lactobacillus* (94% each), *Ruminococcus* (88%) and *Treponema* (65%). NHPs had the highest prevalence of *Prevotella*, the order *Clostridiales* and *Ruminococcus* (100%) followed by *Lachnospiraceae* (96%), *Bacteroidales*_unclassified (92%) *Treponema* (84%) and S24-7 (80%). As anticipated, the human gut microbiota demonstrated high prevalence of OTUs from *Bacteroides*, *Clostridiales_unclassified*, *Ruminococcaceae_unclassified* and *Lachnospiraceae_unclassified* (100%) followed by *Rikenellaceae*_*unclassified* (88%), *Parabacteroides* (80%) and *Faecalibacterium* (76%). The taxa detected in more than 95% samples in each species are presented in Figure 4C.

### Significant Host Species-Specific Differences Are Seen in the Fecal Levels of SCFAs and Lactate

**FIGURE 5:**
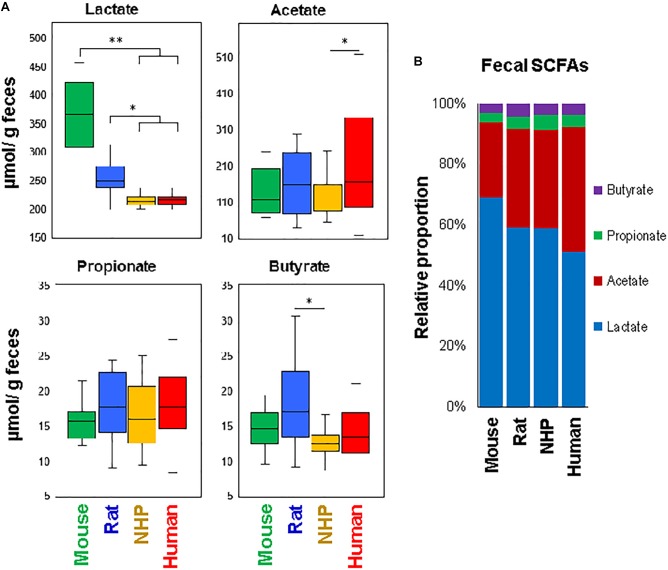
Fecal levels of major short-chain fatty acids in mice, rats, non-human primates (NHP), and human subjects. **(A)** Box-plots showing the concentration of lactate, acetate, propionate, and butyrate in the feces of mice, rats, non-human primates, and human subjects. **(B)** Mean relative proportion of lactate, acetate, propionate, and butyrate (proportionate to the sum of four organic acids) in the feces of mice, rats, non-human primates, and human subjects. ^∗^*P* < 0.05; ^∗∗^*P* < 0.001.

To approximate the similarities or differences in the intestinal organic environment between these four species, we also measured the fecal levels of lactate and major SCFAs, i.e., acetate, propionate, and butyrate (the primary metabolites of gut microbes, especially in the lower part of the intestinal tract), as illustrated in Figure 5. Mice and rats demonstrated higher lactate concentration compared to NHPs and humans, with levels being the highest in mice. On the other hand, the fecal concentration of acetate was the highest in humans, propionate appeared to be similar in all species, whereas butyrate was the highest in rats (Figure 5A). In terms of relative proportion of lactate and these SCFAs (proportionate to the sum of all four organic acids), mice feces were predominated by lactate (69%) followed by acetate (25%), propionate and butyrate (3% each) (Figure 5B). Human specimens had similar higher proportion of lactate (52%) and acetate (41%), followed by propionate (4%) and butyrate (3%). Rats and NHPs had equivalent proportion of lactate (59%) and acetate (33%), although the proportions of propionate and butyrate were 4% for each in rats but 5 and 3%, respectively, in NHPs (Figure 5B). The hierarchical heat-map clustering analyses of correlation between SCFAs/lactate and major OTUs also yielded diverse patterns wherein correlations in human and mice samples were somewhat distinct (with correlations being more prominent in mice compared with those in other three species), whereas rats and NHPs demonstrated somewhat similar pattern of correlations (Supplementary Figure S3).

## Discussion

Despite wide use of various animal models, existing information on comparing their gut microbiome composition with that in humans is limited. However, the usefulness of these models depends largely on the similarities between their and human gut microbiome signatures. To our knowledge, this is the first study comparing the gut microbiota composition and the fecal levels of lactate and SCFAs in humans with those in different laboratory animal species including mice, rats, and NHPs. The overall β-diversity of gut microbiota signatures in the four species suggested that the gut microbiota of mice, rats, NHPs and humans harbor many host species-specific unique bacterial signatures but the patterns of the abundance of these clades might be similar to each other (Figure 1). Higher intra-group UniFrac distance metrics within human subjects pointed toward higher inter-individual variation in human gut microbiota compared to other species. This was somewhat anticipated since mice and rats were maintained at three to five per cage and hence had a higher possibility of microbial exchange. Mice were also maintained throughout life on a chow diet, which would lead to greater similarities in microbial taxa than the NHPs who had a more diverse diet history and were fed two different diet formulations (e.g., Western vs. Mediterranean), or the human subjects who were not on a controlled diet. Further, outbred rats were maintained on two different diets (LF or HF), which may explain the higher intra-group beta-diversity relative to mice. Despite the differences in diet, microbiota signatures from rats remain clearly distinguishable from the other three species. NHPs were also housed socially (4 per pen, with adjacent pens) but still displayed inter-individual variance higher than mice and rats but lower than that within humans, maybe due to different diets than rodents or due to different housing environment, physiologies and others factors. This intra-group inter-individual variation could be at least partly attributed to different dietary regimen of these four species. It is well-established that diet is one of the most prominent elements that can shape and influence the gut microbiome composition (David et al., 2014; Carmody et al., 2015). In our study, human subjects must have had high variability and diversity in their diets (detailed dietary information unknown), whereas NHPs were maintained on one of the two different diets while mice and rats were maintained on single diet. The analysis of within-group variability also demonstrated a similar pattern, i.e., humans had the highest inter-individual variability followed by NHPs while rodents had least variance. Nevertheless, this still hints toward somewhat personalized microbiome signatures among large primates including NHPs and humans and might underpin the importance of considering such inter-individual differences while devising microbiome-targeted therapies such as probiotics, prebiotics, fecal transplants, and other strategies. Nevertheless, these analyses (Figure 1) as well as the measurement of classical hierarchical clustering based on Euclidean similarity and distance indices (Supplementary Figure S4), demonstrate that the gut microbiota in humans was closer to NHPs than to mice and rats, while among rodents, mice appeared to be closer to humans than rats. The analysis of α-diversity indices also indicated that the microbiota in humans might be represented mainly by fewer predominant taxa whereas NHPs might harbor many groups in relatively high abundance.

Mice and rats (particularly those used in the present study) are exclusive herbivores (and coprophagic) whereas humans can be herbivores, carnivores and omnivores, based on ethnicity, geography, culture, and traditions. In addition, there are large anatomical and physiological differences particularly in the gastrointestinal tract which could govern the capacity to which particular microbial species colonize the gut and hence should be considered while interpreting data from these models (Hugenholtz and de Vos, 2018). For instance, the forestomach of mice and rats is used primarily for food storage and has little to no secretory activity (Ghoshal and Bal, 1989) and hence is predisposed to be layered with biofilm comprising strains of various adherent gram-positive bacteria including *Lactobacillus* or other species (Tannock et al., 1994; Benson et al., 2010). Because these bacteria are known to be able to descend from the forestomach and can reach into cecal and fecal bacterial populations (Walter, 2008), this might explain the higher levels of *Lactobacillaceae* and *Lactobacillus* (as well as the lactate) in mice and rats compared to NHPs and humans as seen in the present study. However, bacterial strains found in mice and rats or other rodents can be very different from those found in the NHPs and humans (Frese et al., 2011). In addition, dissimilarities in dietary patterns and circadian rhythms between rodents and humans can also underlie these microbiome differences (Hugenholtz and de Vos, 2018). Mice and rats eat almost incessantly during the night cycle, whereas humans eat intermittently during the daytime especially when the stomach gets somewhat empty. Therefore, in the rodent stomach, freshly consumed diet components are incessantly blended with gastric fluids thereby diluting the bolus and gastric acids (Schwarz et al., 2002), which may lead to relatively high pH in the rodent stomach thus allowing bacterial biofilms (Bik et al., 2006). Notably, this may also explain to some extent the higher fecal concentration of acetic acid in humans compared with mice and rats; although higher acetate in humans may also be attributed to the predominant abundance of *Bacteroides*. Also, the higher abundance of *Bacteroides*, *Bifidobacterium*, and *Akkermansia* in humans particularly versus mice and rats could be at least partly ascribed to higher thickness and growth rate of mucus in humans relative to rodents (Gustafsson et al., 2012), since many species belonging to *Bacteroides*, *Akkermansia*, and *Bifidobacterium* are potential mucus-degraders (Wacklin et al., 2014) and are known to be able to often carry adhesins through which they can bind mucins (Sommer et al., 2014). This might also include differences in several other mucus-associated bacteria between humans and rodents. Intriguingly, the abundance of *Akkermansia* was relatively comparable between mice and humans, thus suggesting that the proliferation of this or similar bacterial groups may be similar despite differences in mucus composition and concentration.

Except for the higher abundance of *Actinobacteria* in humans, the phylum level microbiota configuration seemed to be similar in mice and humans, although the *Firmicutes* : *Bacteroides* ratio was slightly higher in humans, as reported previously (Ley et al., 2006a,b, 2008; Rawls et al., 2006; Wos-Oxley et al., 2012). Such differences in the *Firmicutes* : *Bacteriodetes* ratio between rodents and humans could imply a limitation to the use of these animal models since the populations of major groups belonging to these phyla might not imitate the ratio typically found in a healthy human gut. However, these models might still be useful to study human gut-related maladies, particularly given that the proportions of *Firmicutes* are usually found to be distorted in patients with inflammatory bowel disease including Crohn’s disease, ulcerative colitis, and infectious colitis (Manichanh et al., 2006; Sokol et al., 2009). Nevertheless, this might indicate that qualitatively the microbiota of mice and humans may look alike but quantitatively is very different. Mice harbored higher abundance of the phylum *Deferribacteres*, which were detected only in minute amounts in humans, rats, and NHPs. This was also reflected at the genus-level in terms of higher abundance of *Mucispirillum*, one of the major member of this phylum and also a potential colonizer of the mucus layer in mice (Robertson et al., 2005).

We also found higher abundance of the genera *Faecalibacterium* and *Dialister* in humans than in other three species. Previous reports have also demonstrated similar differences particularly between human and murine microbiota, but the prevalence of these genera may also differ between different rodent strains. Further larger and all-inclusive studies should be able to validate and interpret such differences between human and animal microbiome. We selected C7BL/6 because this is the most-widely used strain for understanding human microbiome dynamics; however, their microbiome might be different from that in other mouse strains. For example, the abundance of both *Akkermansia* and *Lactobacillus* has been reported to differ between C57BL/6, BALB/c, and NOD mice (Xiao et al., 2015). However, data comparing the microbiomes between different mouse strains are still limited and future studies examining the gut microbial similarities and dissimilarities between these strains would facilitate understanding the phylogenetic makeup in these strains and interpreting it with reference to the human gut microbiome.

Non-human primates also demonstrated a unique spectrum of gut bacterial consortia that differed not only from mice and rats but also from humans. Indeed, compared to humans, NHPs are characterized by many unique physiological characteristics, e.g., smaller brain size, larger gut size, lower fat deposition and a different spectrum of intestinal Toll-like receptors (Aiello and Wheeler, 1995; Kargman et al., 1996; Wlasiuk and Nachman, 2010), and hence can be expected to have a different gut microbiome. Given that the microbiome development is associated with the host’s diet, metabolism and physiology, it is highly plausible that evolutionary shifts in primates’ diet and physiology and exposures to environmental compounds also drove dramatic changes in the inherent gut microbiome consortium. In one of our recent reports, we demonstrated remarkable shifts in NHP microbiota following Mediterranean- or western-style dietary intervention wherein some of the changes were similar to those generally seen in humans consuming these diets (Nagpal et al., 2018). Notably, the gut microbiome composition in NHPs is more similar to that in human primates than that in other animals (Ley et al., 2008). For instance, the human gut is inhabited by bacteria belonging to nine phyla: *Firmicutes* and *Bacteroides* (most abundant and predominant phyla), *Actinobacteria, Fusobacteria, Proteobacteria, Verrucomicrobia, Cyanobacteria, Spirochaetes* and *VadinBE97* (Backhed et al., 2005; Ley et al., 2006b). We identified *Bacteroidetes, Firmicutes* (most abundant phyla), *Proteobacteria, Actinobacteria, Verrucomicrobia, Fibrobacteres, Cyanobacteria, Spirochaetes*, and *Tenericutes* as the nine major phyla in NHPs (Nagpal et al., 2018). Hence, despite considerable qualitative and quantitative differences in bacterial abundance, investigation of specific bacterial clades colonizing the NHPs gut may still provide important information about the features of these bacteria in the host gut.

Our study has some limitations. First, the current study included only a single strain each of mice, rats, and primate species. Future work should include other strains (and heterogeneous sets) of mice and rats, or other animal species (hamsters, guinea pigs, rabbits, swine, and others) commonly used as models. In the current study, all mice and rats were males, NHPs were all females, whereas human subjects included both males and females (although, no gender based differences observed, Supplementary Data Sheet 1). Another limitation is the difference in the dietary intake of difference groups, which could not be controlled in this study. Although controlling for dietary patterns in studies involving different animal species is practically not feasible but future studies may try to control for as much as possible at least in terms of overall macronutrient/calorie composition. Also, the current study measured only major SCFAs and hence the data on other fecal metabolites and SCFAs (e.g., valerate, succinate, and others) remain unknown. Nevertheless, the study also has several strengths. For example, the number of subjects in each species group is adequate, particularly considering that majority of animal studies are performed on more than 17 animals and humans. Another advantage is that the study was conducted within the same facility wherein all the experiments were performed by the same laboratory personnel and by using the same protocols of sample collection and processing, DNA extraction, library preparation, 16S sequencing as well as the measurement of lactate and SCFAs. To our knowledge, this is the first study comparing the gut microbiota composition and the fecal levels of lactate and SCFAs in humans to those in some of the most-widely used animal models, particularly in the same setting and using same experimental procedures. The results demonstrate that despite large differences in the relative abundance of many bacterial clades, a considerable fraction of not only major phyla but also many common genera are shared extensively between mice, rats, NHPs, and humans. The data provide important information about host species-specific gut microbiota signatures in these species and should be valuable for prospective studies focused on investigating various aspects of gut microbiome in health and disease. While beyond the scope of this work, it will be important to determine host species-specific differences in other animal species using 16S metagenomic approach as well as transcriptomic, metabolomic, and proteomic approaches.

## Availability of Data and Materials

The raw data of alpha-diversity indices, the 16S sequencing OTU proportional abundance, and the fecal levels of lactate and SCFAs are deposited in the supplemental data section (Supplementary Table S1).

## Ethics Statement

All protocols related to the subjects involved in the study have been reviewed and approved by the Institutional Animal Care and Use Committee and the Institutional Ethical Committee of the Wake Forest School of Medicine and National Diabetes and Digestive and Kidney Diseases, National Institutes of Health. All experiments and samplings were carried out in accordance with ethical and biosafety protocols approved by the Institutional guidelines.

## Author Contributions

RN measured microbiome, data analysis, interpretations, and wrote the first draft of the manuscript. SW measured lactate and SCFAs. LSW and OS designed rat studies, provided rat feces, and supported financially for microbiome sequencing for rat fecal 16S rRNA analyses. SCh and SCr conducted human studies and coordinated in collecting human feces from subjects enrolled in respective studies. CS and TR coordinated in NHP colony management and sample collection. DM coordinated in acquiring mice and in study designs. HY conceived idea, assisted with the data analysis and interpretations, and revised drafts of manuscript. All the authors critically reviewed and edited manuscript.

## Conflict of Interest Statement

The authors declare that the research was conducted in the absence of any commercial or financial relationships that could be construed as a potential conflict of interest.

## Footnotes

1 https://www.r-project.org/
