# Supplementary material for: Comparative Microbiome Signatures and Short-Chain Fatty Acids in Mouse, Rat, Non-human Primate, and Human Feces
Source: Front Microbiol. 2018 Nov 30;9:2897. doi: 10.3389/fmicb.2018.02897 (PMC6283898; doi:10.3389/fmicb.2018.02897)

Supplementary material

Suppl. Fig. 1. Mean relative abundance of major bacterial classes (a) and order (b) detected in the gut microbiome of mice, rats, non-human primates, and human subjects.

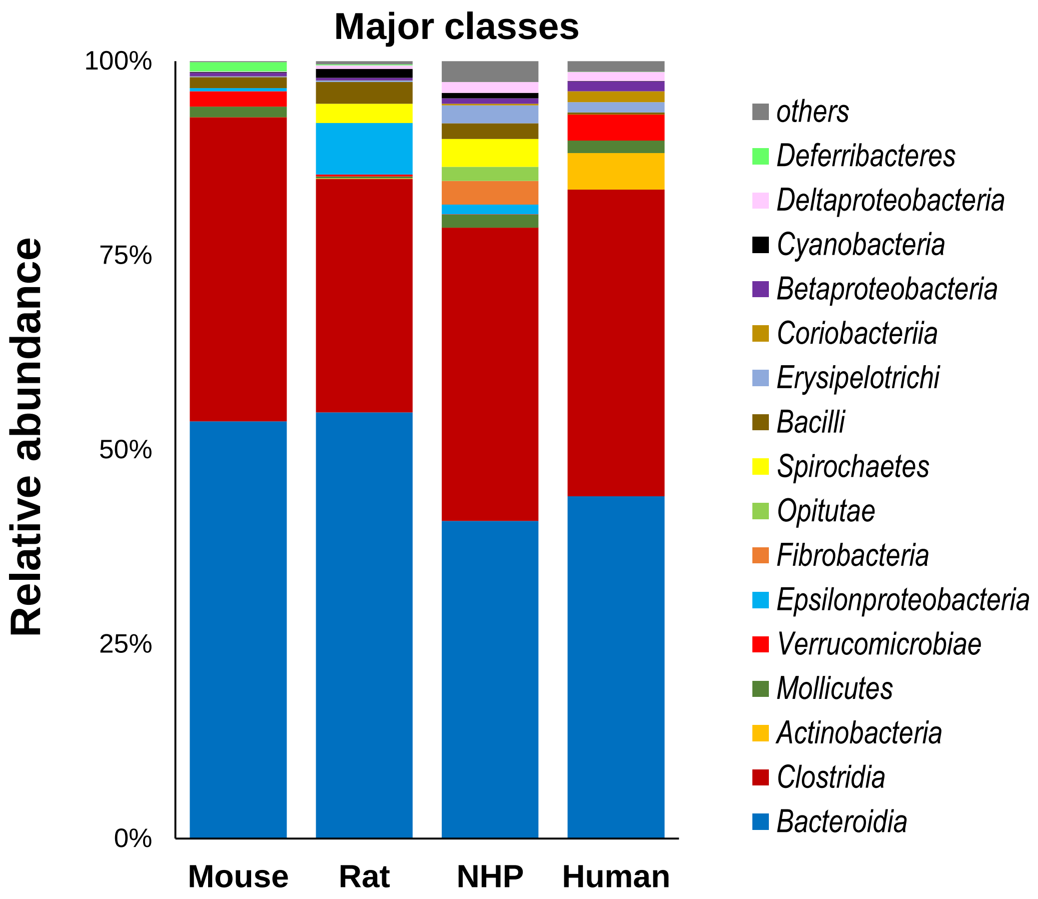

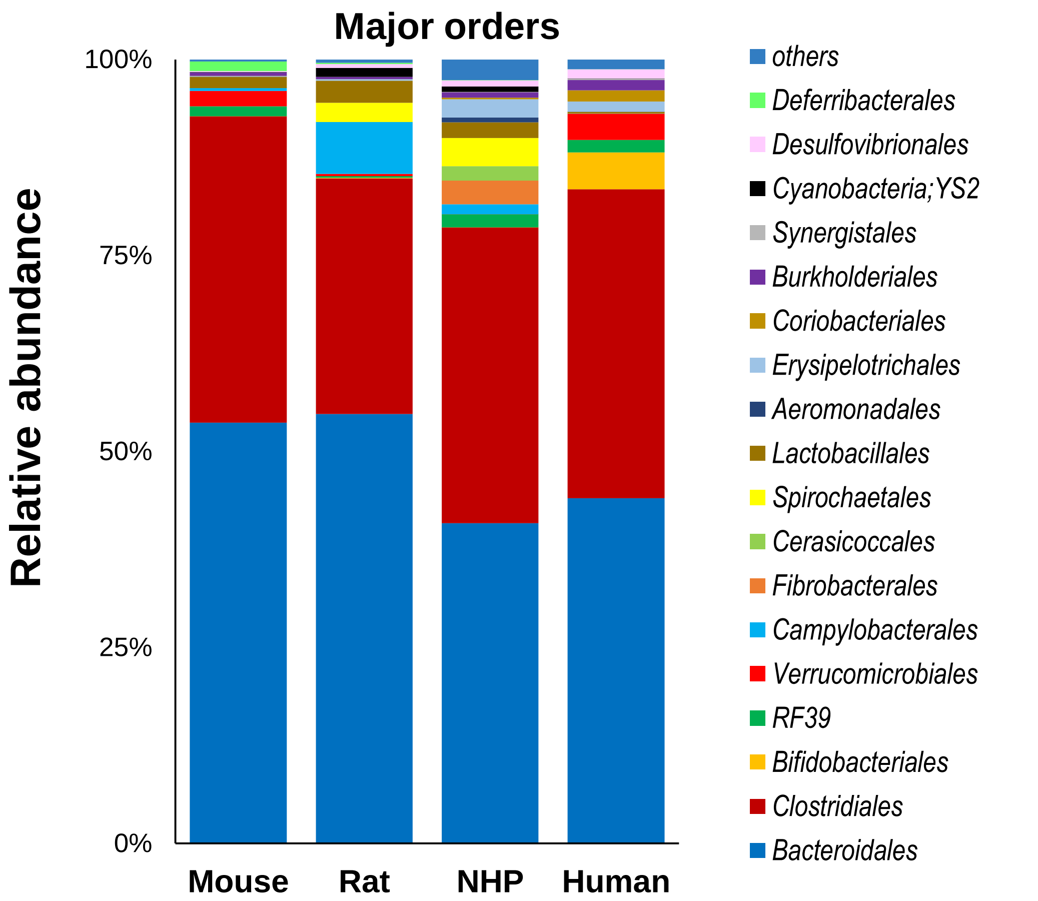

Suppl. Fig. 2. Linear discriminant analysis (LDA) effect size (Lefse) cladogram representing the unique bacterial signatures identified in mice, rats, non-human primates, and human subjects.

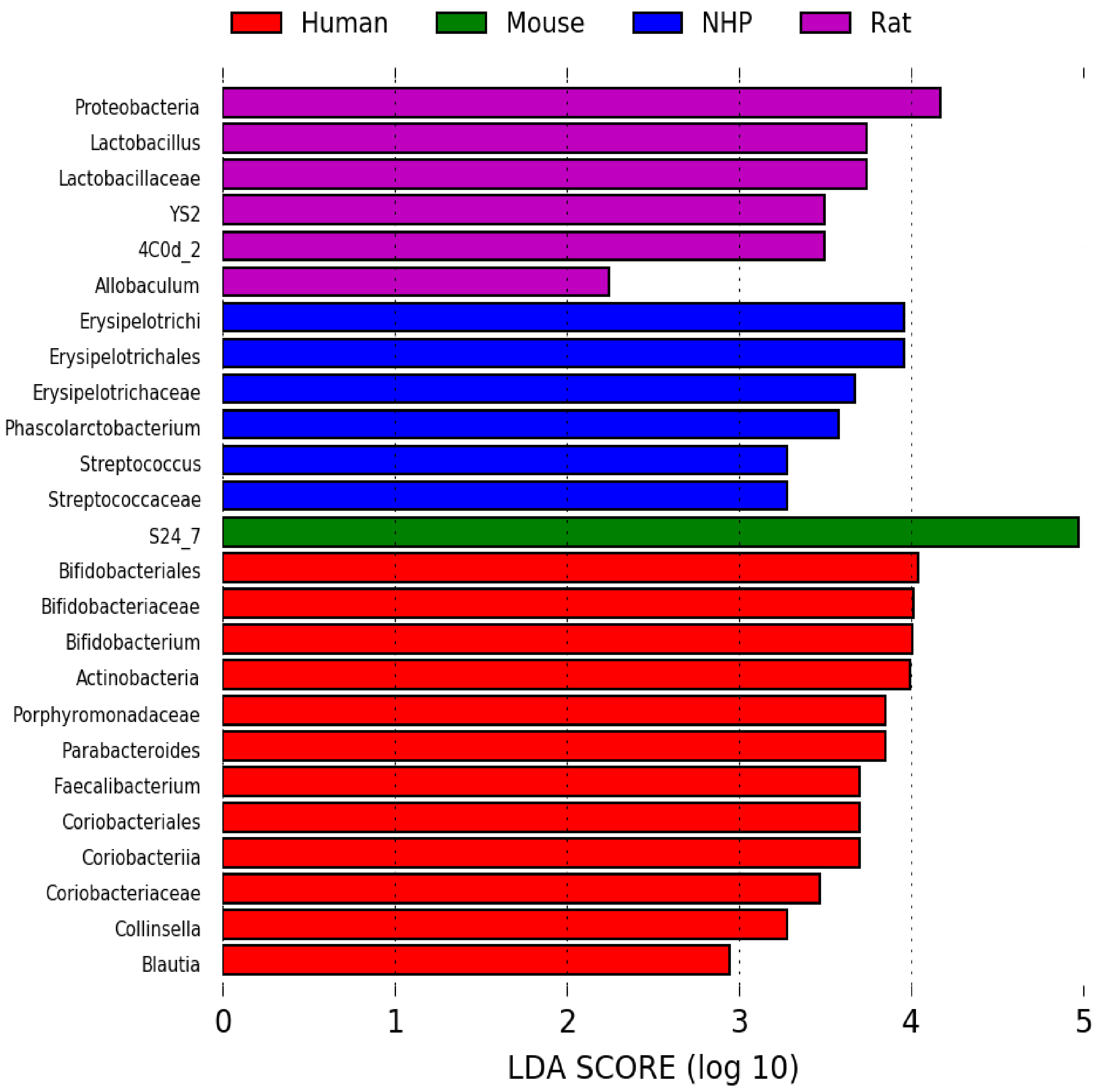

Suppl. Fig. 3. Heat-map representing the clustering patterns of correlation between the abundance of major gut bacterial OTUs and the fecal levels of major short-chain fatty acids in mice, rats, non-human primates, and human subjects.

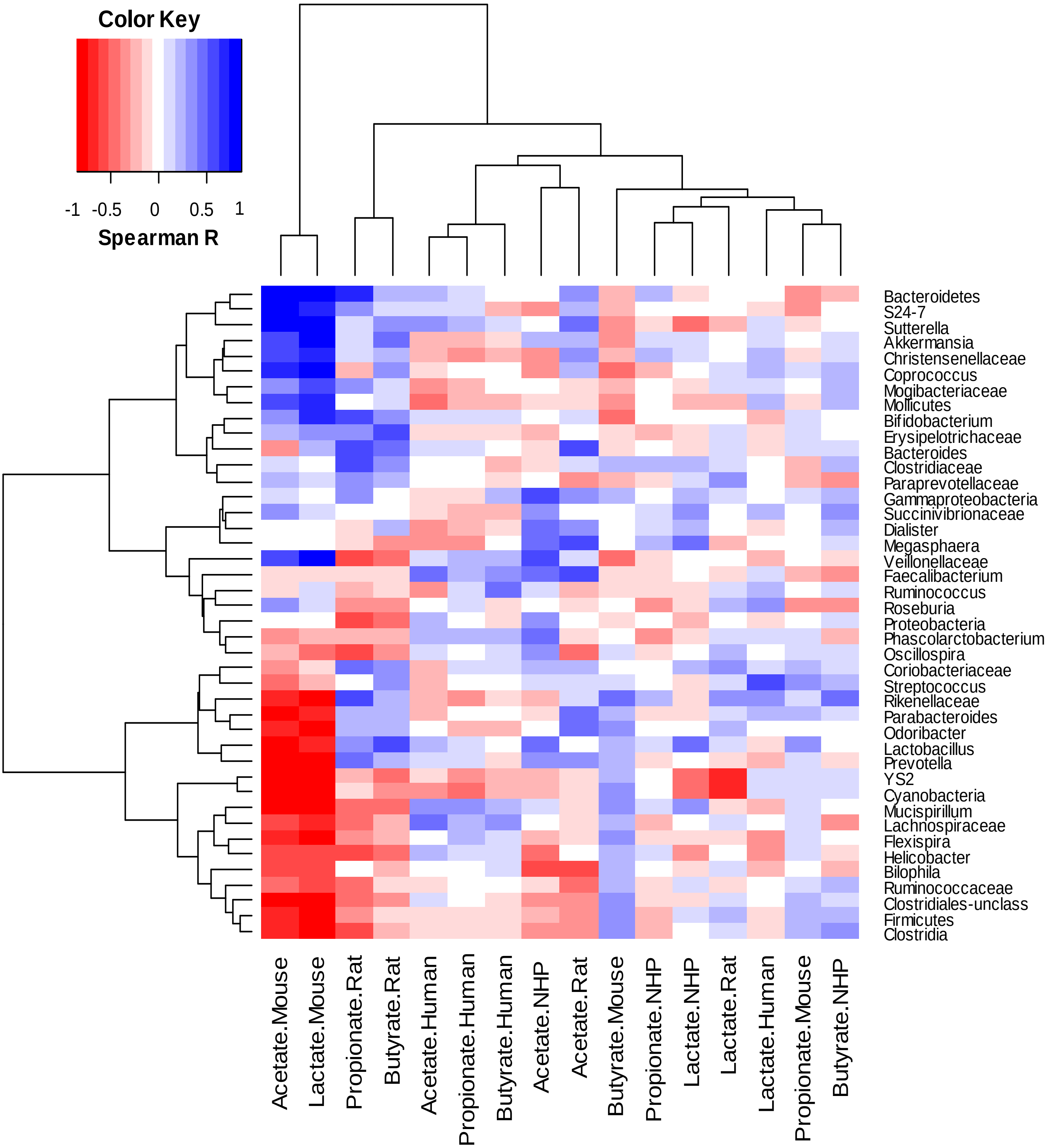

Suppl. Fig. 4. Hierarchical clustering representing the Euclidean similarity index (a) and distance (b) between the gut microbiome of mice, rats, non-human primates, and human subjects.

(a)

Neighbor Joining Clustering: Euclidean Similarity Index

(b)

Classical Hierarchical clustering: Euclidean distance

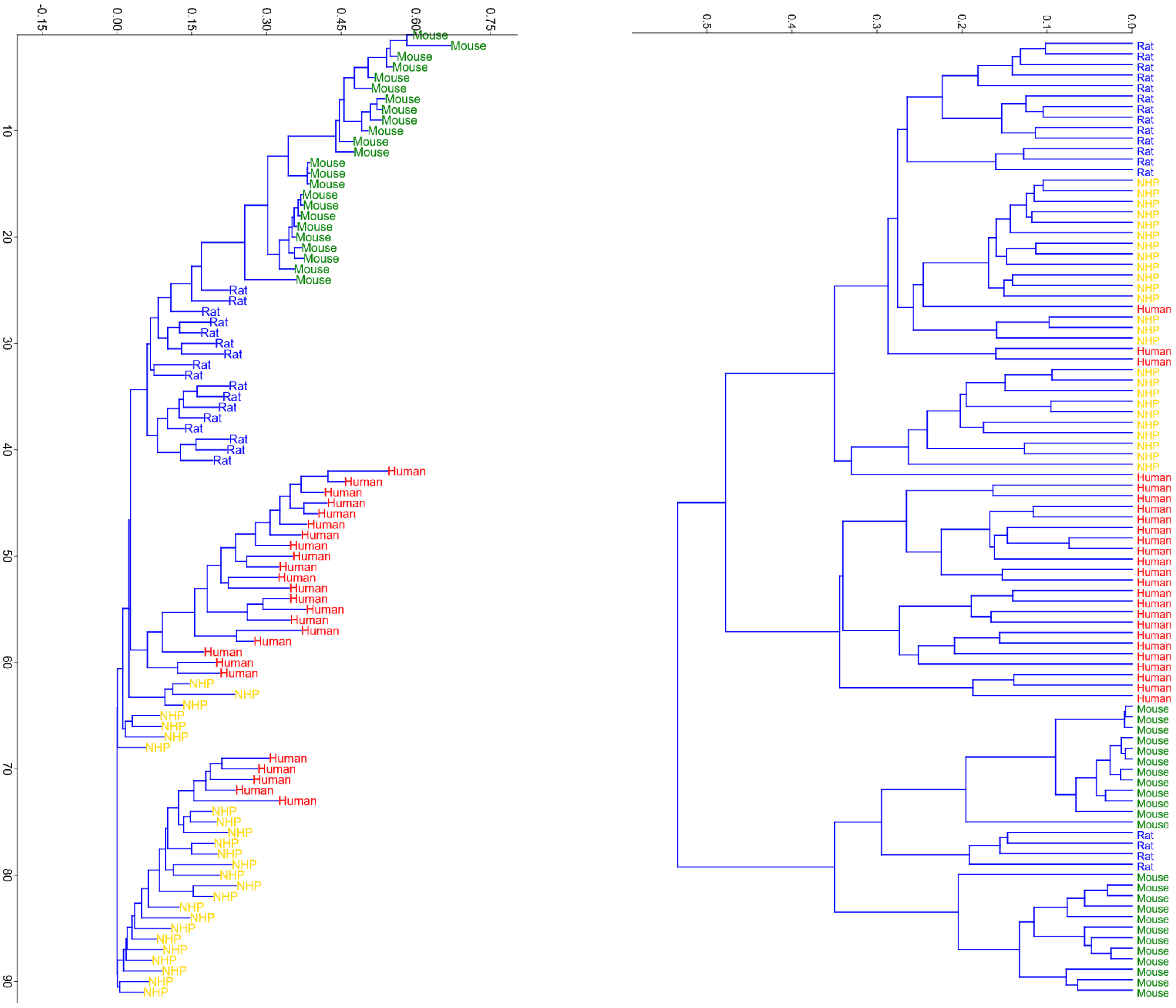

Supplement: Supplementary file 2 [file Data_Sheet_1.pdf]
