# Supplementary material for: Comparative Microbiome Signatures and Short-Chain Fatty Acids in Mouse, Rat, Non-human Primate, and Human Feces
Source: Front Microbiol. 2018 Nov 30;9:2897. doi: 10.3389/fmicb.2018.02897 (PMC6283898; doi:10.3389/fmicb.2018.02897)
Supplement: Supplementary file 3 [file Data_Sheet_2.pdf]

Supplemental material:

Comparison of gut microbiome and organic acids in male vs. female human subjects included in the present study.

Beta-diversity of the gut microbiome in male vs. female human subjects included in the present study.

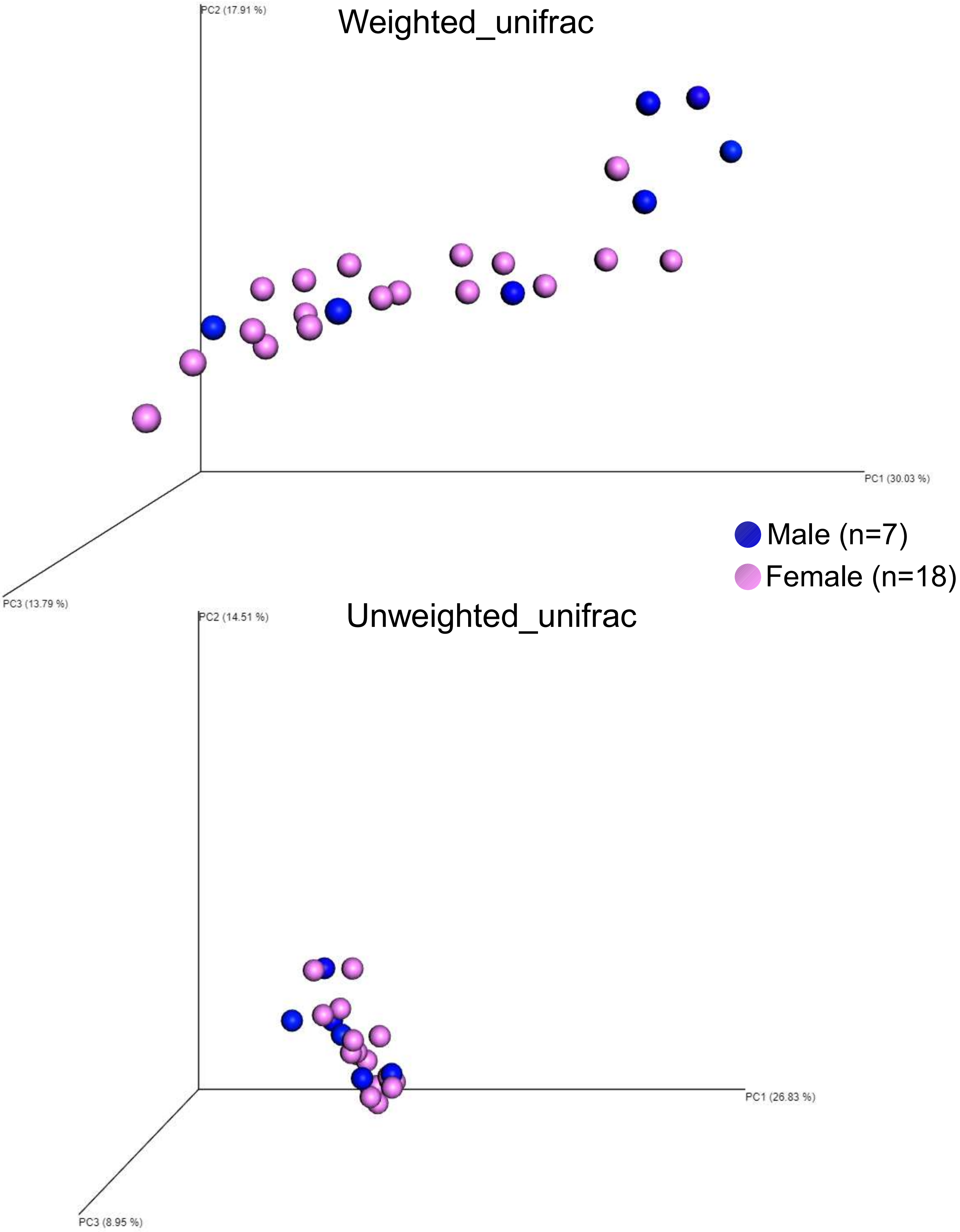

Alpha-diversity of the gut microbiome in male vs. female human subjects included in the present study.

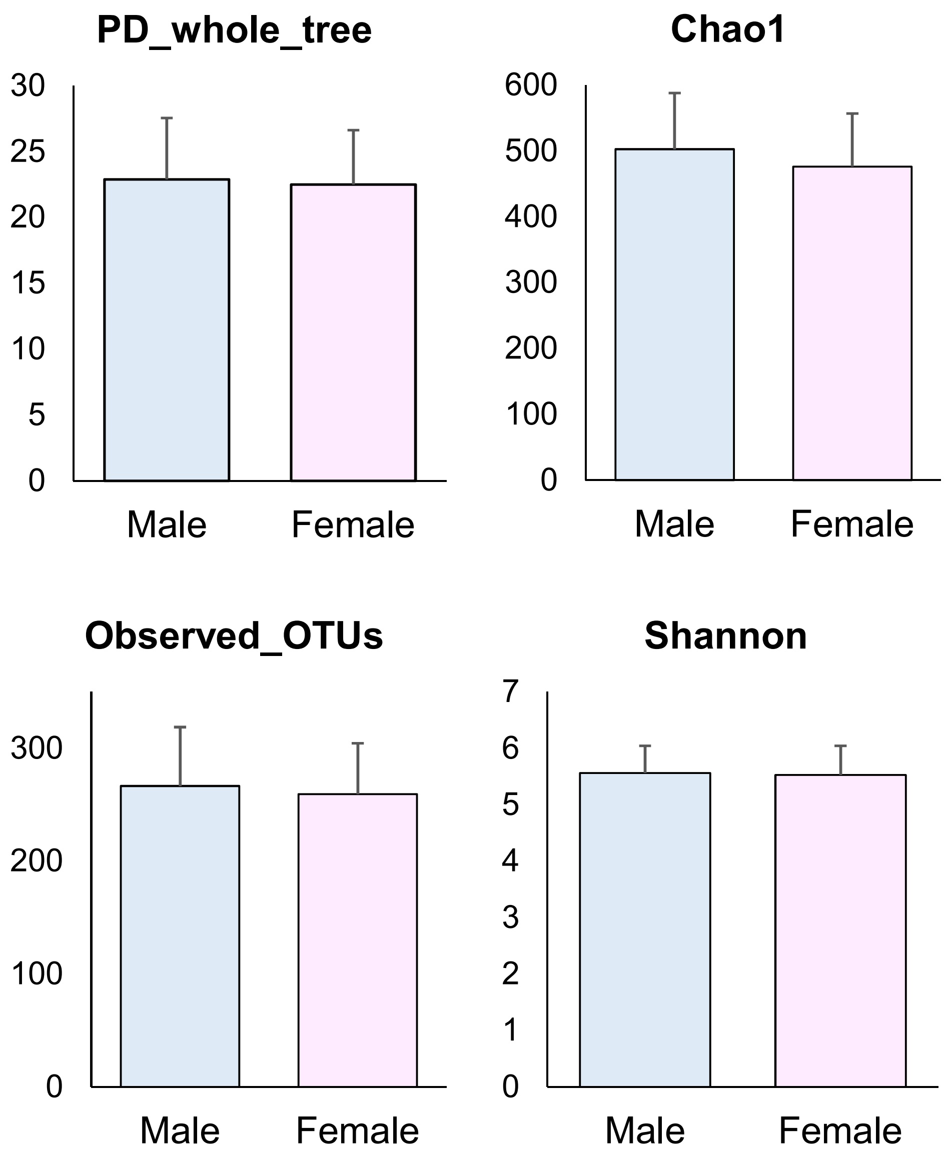

No significant difference.

Relative abundance of major phyla detected in male vs. female human subjects included in the present study.

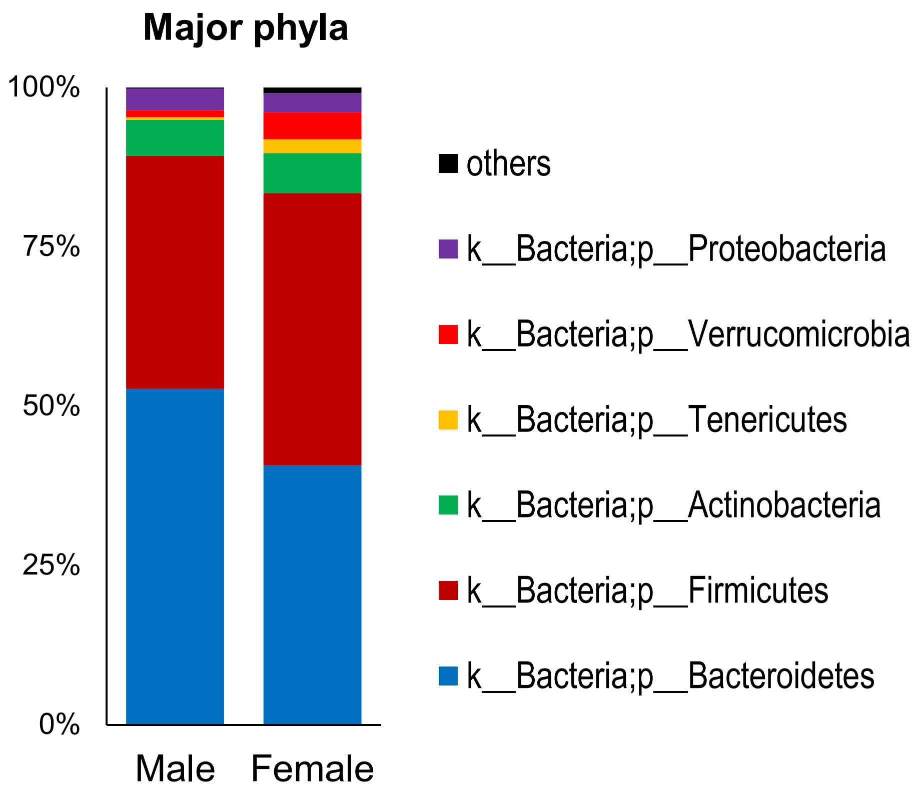

No significant difference.

Relative abundance of major bacterial families and genera detected in male vs. female human subjects included in the present study.

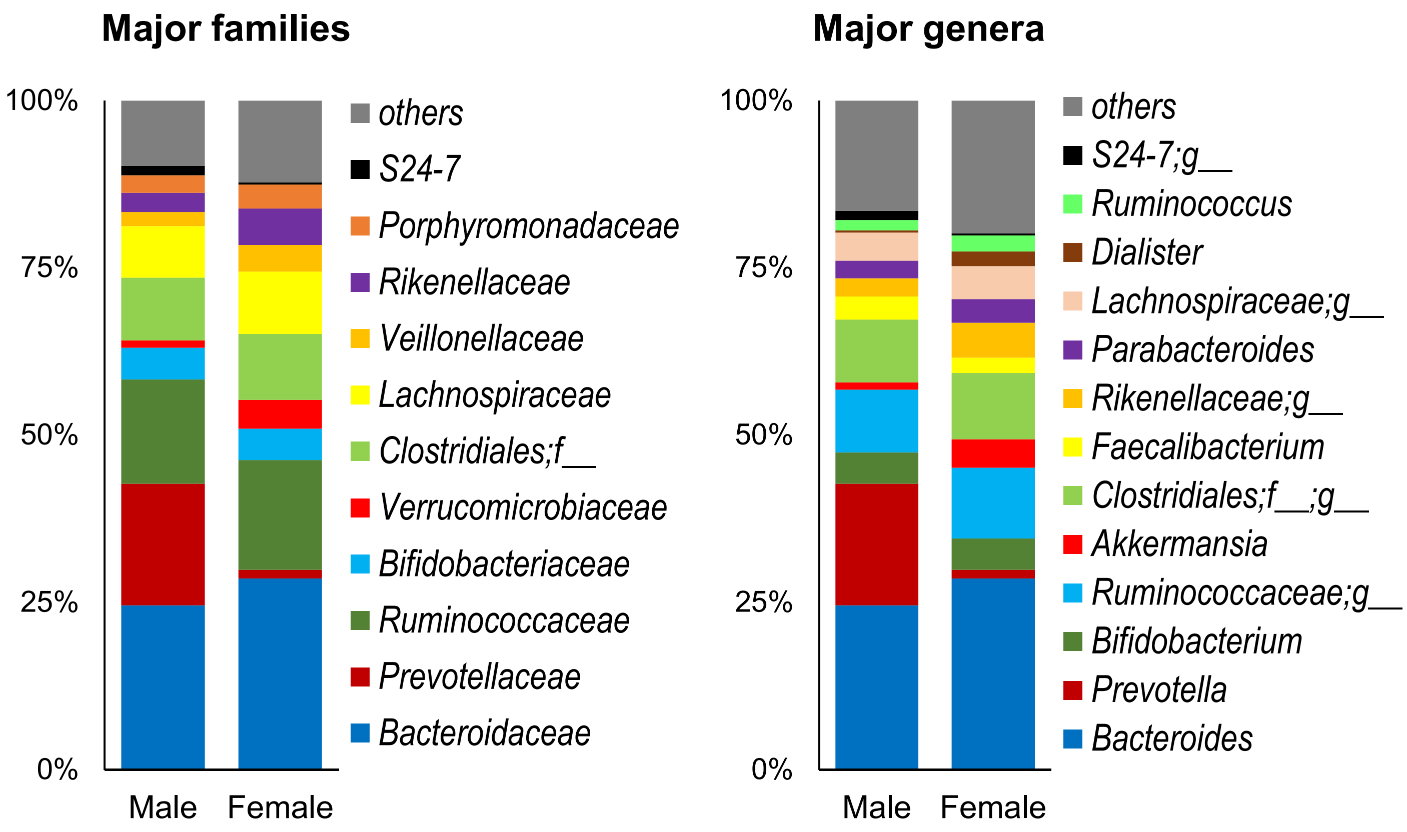

Only *Prevotellaceae* and *Prevotella* are significantly different.

Fecal concentration of lactate and major short-chain fatty acids in male vs. female human subjects included in the present study.

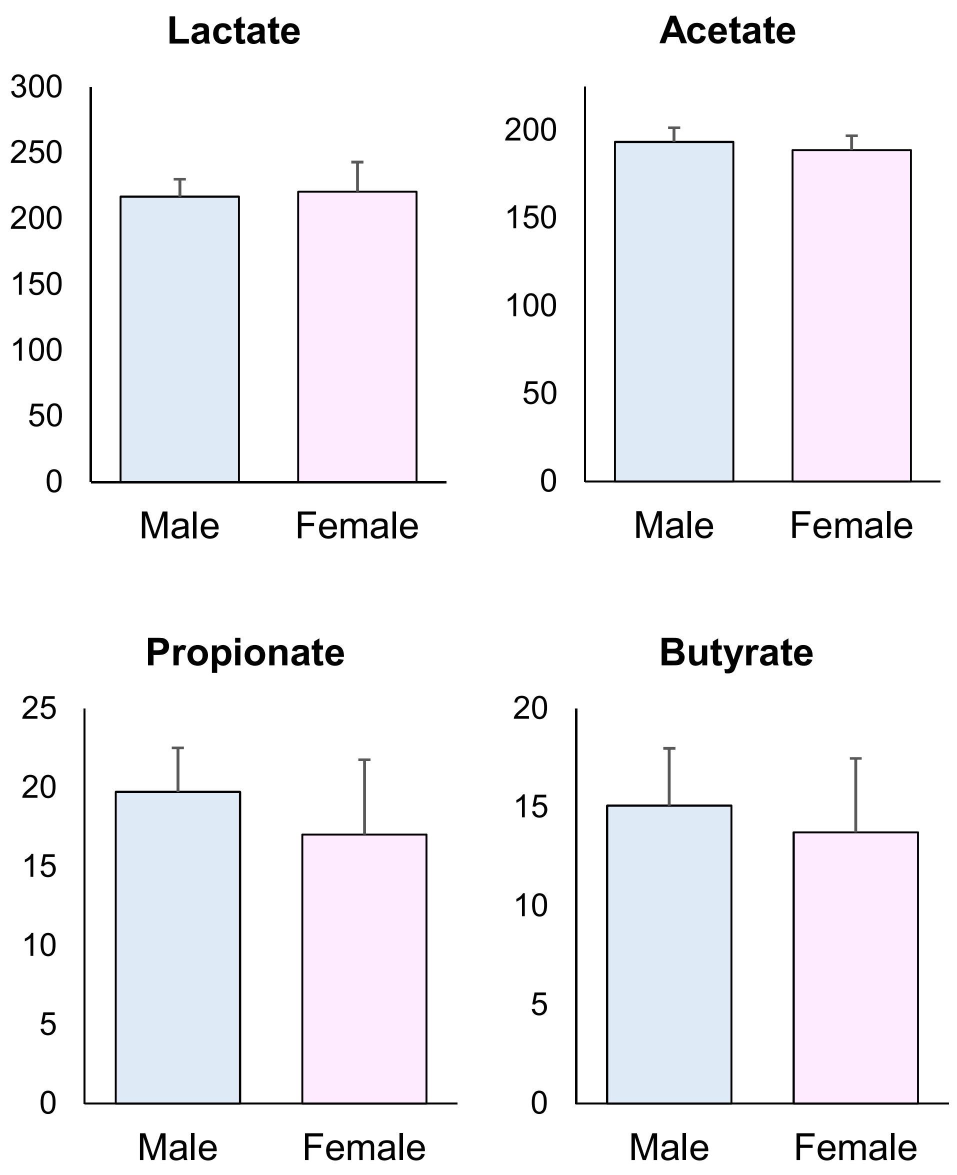

No significant difference.
